# Supplementary material for: Digital traces of brain drain: developers during the Russian invasion of Ukraine
Source: EPJ Data Sci. 2023 May 15;12(1):14. doi: 10.1140/epjds/s13688-023-00389-3 (PMC10184088; doi:10.1140/epjds/s13688-023-00389-3)
Supplement: Supplementary file 1 — Supplementary information (PDF 113 kB) [file 13688_2023_389_MOESM1_ESM.pdf]

Appendix for: Digital Traces of Brain Drain

Johannes Wachs [1,2,3]

[1] Corvinus University Budapest
[2] Centre for Economic and Regional Studies, Hungary
[3] Complexity Science Hub Vienna
Correspondence to: johannes.wachs@uni-corvinus.hu

Overview

In this appendix we report a variety of robustness checks. We filter the original dataset to include only those developers with a greater number of commits. We also rerun the network analysis of the main article focusing on those repos with relatively few contributors.

Filtering on number of commits

Here we report the results of our analysis when filtering for developers with at least 200 or 500 commits in the 2019-2020 period (vs. 100 in the primary article). Unless otherwise noted, differences in means are significant at $p < 0.001$ according to Mann-Whitney U tests. The purpose of this robustness test is to observe the consistency of the results among smaller, even more active subpopulations of the full data sample. Under both alternatives our results remain substantively unchanged compared with the results presented in the text.

At least 200 commits In Table 1 we report the change in account location status comparing 2021 vs November 2022, filtering at the threshold of 200 commits. In Table 2 we report the destinations of leavers. In both cases we find results highly similar to the main results of the paper. Regarding differences in activity, we again find that leavers were previously more active than remainers (medians: 409 vs 346, means: 711 vs 547). Among developers locatable in November 2022, 12.6% left and account for 15% of all commits. Leavers have more collaboration network connections (mean 3.6 vs 1.4) and are more likely to be in the largest connected component of the 2019-2020 Russian collaboration network (17.7% vs 9.7%). Repeating the exercise of removing leavers versus removing random subsets of developers of the same size results in a similar Z-score for the entropy of the collaboration network connected component sizes: 5.2 (vs 5.6 in the main article).

Country	# of Developers	Profile Deleted (A)	Invalid/No Loc. (B)	% New Country (C)	A+B+C
Russia	6866	2.9%	15.2%	12.6%	30.8%
Belarus	902	3.9%	10.0%	20.0%	33.8%
Ukraine	2918	3.9%	2.7%	4.7%	11.2%
Estonia	270	1.5%	2.6%	8.1%	12.3%
Latvia	162	1.2%	5.0%	3.1%	9.4%
Lithuania	306	2.0%	2.0%	2.9%	6.9%
Poland	4009	2.6%	2.5%	2.4%	7.5%
Czechia	1472	1.8%	2.2%	3.5%	7.5%
Slovakia	284	1.8%	3.6%	5.6%	11.0%
Bulgaria	788	2.0%	1.7%	2.0%	5.7%
Romania	790	2.3%	3.5%	3.4%	9.2%
Hungary	751	1.2%	3.8%	3.9%	8.9%
Serbia	413	2.9%	4.0%	4.6%	11.5%

Table 1: Data on developer account deletion, location removal, and emigration filtered on developers with at least 200 commits (vs 100 in primary analysis) in November 2022.

Russian	Count	Pct.	Belarus	Count	Pct.	Ukraine	Count	Pct.
United States	100	12%	Poland	79	44%	United States	26	19%
Germany	84	10%	Georgia	18	10%	Poland	16	12%
Georgia	61	7%	Lithuania	14	8%	Germany	13	10%
Netherlands	60	7%	United States	12	7%	Canada	10	7%
Armenia	51	6%	Estonia	6	3%	Russia	10	7%
Cyprus	47	5%	Spain	5	3%	Czechia	8	6%
United Arab Emirates	46	5%	Germany	4	2%	United Kingdom	7	5%
Türkiye	45	5%	France	4	2%	Netherlands	5	4%
Serbia	34	4%	Norway	4	2%	Sweden	4	3%
United Kingdom	30	3%	United Kingdom	3	2%	Spain	4	3%
Kazakhstan	23	3%	Czechia	3	2%	Italy	4	3%
Finland	20	2%	Ukraine	3	2%	Portugal	3	2%
Israel	17	2%	Russia	3	2%	Norway	2	1%
Poland	15	2%	Latvia	3	2%	Slovakia	2	1%
Czechia	14	2%	Netherlands	2	1%	China	2	1%
Montenegro	14	2%	Portugal	2	1%	Switzerland	2	1%
Canada	14	2%	Armenia	1	1%	France	2	1%
Indonesia	12	1%	Türkiye	1	1%	Finland	2	1%
Switzerland	12	1%	Sweden	1	1%	Ireland	2	1%
Estonia	10	1%	United Arab Emirates	1	1%	Türkiye	1	1%

Table 2: Destination countries for developers, considering only those with at least 200 commits (vs 100 in primary analysis).

Country	# of Developers	Profile Deleted (A)	Invalid/No Loc. (B)	% New Country (C)	A+B+C
Russia	2244	2.4%	19.2%	15.6%	37.2%
Romania	303	1.7%	2.7%	3.3%	7.7%
Ukraine	882	2.4%	3.2%	5.9%	11.4%
Lithuania	97	3.1%	1.1%	4.1%	8.3%
Latvia	60	1.7%	6.9%	6.7%	15.2%
Slovakia	103	1.9%	4.0%	7.8%	13.7%
Poland	1359	2.1%	2.4%	2.7%	7.2%
Czechia	697	1.7%	2.5%	3.0%	7.2%
Estonia	97	0.0%	3.1%	5.2%	8.2%
Hungary	274	0.7%	3.7%	4.0%	8.5%
Bulgaria	301	3.0%	2.8%	2.7%	8.4%
Belarus	264	3.4%	11.5%	25.8%	40.6%
Serbia	109	2.8%	5.7%	1.8%	10.3%

Table 3: Data on developer account deletion, location removal, and emigration filtered on developers with at least 500 commits (vs 100 in primary analysis) in November 2022.

At least 500 commits In Table 3 we report the change in account location status comparing 2021 vs November 2022, filtering at the threshold of 500 commits. In Table 4 we report the destinations of leavers. In both cases we find results highly similar to the main results of the paper. Regarding differences in activity, we again find that leavers were previously more active than remainers (medians: 861 vs 782, means: 1,295 vs 1,124). Among developers locatable in November 2022, 15.6% left and account for 16.9% of all commits. Leavers have more collaboration network connections (mean 3.2 vs 1.1) and are more likely to be in the largest connected component of the 2019-2020 Russian collaboration network (16.3% vs 9.2%). Repeating the exercise of removing leavers versus removing random subsets of developers of the same size results in a smaller but still statistically significant ($p < .05$) Z-score for the entropy of the collaboration network connected component sizes: 2.2 (vs 5.6 in the main article).

Russian	Count	Pct.	Belarus	Count	Pct.	Ukraine	Count	Pct.
United States	36	10%	Poland	31.0	46%	United States	12	23%
Germany	33	9%	Lithuania	6.0	9%	United Kingdom	5	10%
Netherlands	33	9%	Georgia	5.0	7%	Poland	5	10%
Cyprus	24	7%	United States	4.0	6%	Germany	4	8%
United Arab Emirates	23	7%	Germany	4.0	6%	Czechia	3	6%
Armenia	19	5%	Estonia	3.0	4%	Canada	3	6%
Türkiye	19	5%	Latvia	2.0	3%	Italy	2	4%
Serbia	12	3%	Norway	2.0	3%	Portugal	2	4%
Georgia	11	3%	United Kingdom	2.0	3%	Russia	2	4%
Kazakhstan	11	3%	Ukraine	1.0	1%	Slovakia	2	4%
United Kingdom	10	3%	Spain	1.0	1%	Sweden	2	4%
Switzerland	9	3%	Armenia	1.0	1%	Indonesia	1	2%
Czechia	8	2%	Malaysia	1.0	1%	Latvia	1	2%
Finland	8	2%	Kazakhstan	1.0	1%	Malta	1	2%
Canada	7	2%	Czechia	1.0	1%	Georgia	1	2%
Indonesia	7	2%	Cyprus	1.0	1%	France	1	2%
Thailand	6	2%	Canada	1.0	1%	Spain	1	2%
Israel	6	2%	Uzbekistan	1.0	1%	Switzerland	1	2%
Lithuania	5	1%	-	-	-	Estonia	1	2%
Estonia	4	1%	-	-	-	Denmark	1	2%

Table 4: Destination countries for developers, considering only those with at least 500 commits (vs 100 in primary analysis).

Network Analysis: Excluding repos with many contributors

Here we report the results of our network analysis rerun after excluding repos with more than 10 contributors, considered globally (i.e. not restricted to Russia-based developers). The purpose of this analysis is to consider situations which can more confidently be considered as collaborations: in this subset of repos developers are much more likely to directly interact with one another. When considering those repos with at most 10 contributors, we find that Russia leavers have more connections than remainers (mean: 0.71 vs 0.51; stdev: 1.48 vs 1.21). Leavers are also more likely to be in the largest connected component (5.4% are in the LCC vs 3.3% of remainers).
